# Innovative Partnerships to Address Food Insecurity during the COVID-19 Pandemic: The Brighter Bites Produce Voucher Program

**DOI:** 10.3390/ijerph18179175

**Published:** 2021-08-31

**Authors:** Amier Haidar, Christine Markham, Allison Marshall, Ru-Jye Chuang, Meredith Spence, Jennifer Boone, Mike Pomeroy, Rich Dachman, Jaimie N. Davis, Shreela V. Sharma

**Affiliations:** 1 Department of Epidemiology, Human Genetics and Environmental Sciences, Michael & Susan Dell Center for Healthy Living, The University of Texas Health Science Center at Houston (UTHealth) School of Public Health, 1200 Pressler Street, Houston, TX 77030, USA; allison.n.marshall@uth.tmc.edu (A.M.); ru-jye.chuang@uth.tmc.edu (R.-J.C.); shreela.v.sharma@uth.tmc.edu (S.V.S.); 2 Department of Health Promotion and Behavioral Sciences, The University of Texas Health Science Center at Houston (UTHealth) School of Public Health, 1200 Pressler Street, Houston, TX 77030, USA; christine.markham@uth.tmc.edu; 3 Brighter Bites, Houston, TX 77029, USA; meredith.spence@brighterbites.org (M.S.); jenniferboone@utexas.edu (J.B.); mike.pomeroy@brighterbites.org (M.P.); rich.dachman@brighterbites.org (R.D.); 4 Department of Nutritional Sciences, University of Texas, Austin, TX 78723, USA; jaimie.davis@austin.utexas.edu

**Keywords:** produce voucher program, COVID-19, non-profit–for-profit partnerships, vulnerable populations

## Abstract

The purpose of this communication is to describe the Brighter Bites produce voucher program, and its implementation and utilization across Brighter Bites families in four cities in the U.S., during the COVID-19 pandemic. The voucher program was implemented over nine weeks starting April 2020, with up to four USD 25 store-specific produce coupons sent bi-weekly to the homes of each participating Brighter Bites family (USD 100 total/family). Measures included type of produce purchased, amount of voucher that was used, number of vouchers distributed and redeemed by families, and a post-program participant satisfaction survey. Descriptive statistics, including count, frequency, and percent, were computed, both overall and stratified by city. During this time, Brighter Bites distributed a total of over 43,982 vouchers to 12,482 low-income families, with a redemption rate of 60% (at least one voucher redeemed) across all cities. During times of crisis, non-profit–for-profit partnerships, such as the one between Brighter Bites and the grocery retail industry, are feasible, and successful in providing produce to families in need.

## 1. Introduction

SARS COV-2 (COVID-19) was declared a pandemic by the World Health Organization on 11 March 2020 [1]. Throughout the month of March, many states began issuing stay-at-home orders and started implementing social distancing measures, including school closures, work from home policies, and cancellations of public gatherings [2]. These social distancing measures had a significant economic impact, by disrupting food systems and exacerbating the economic, social and health needs among families. Numerous people were left unemployed, food insecure, financially strained, and unable to access healthcare [3,4,5]. In addition, school closures prevented access to free and reduced lunch programs for children [3,4,5].

According to the United States Department of Agriculture (USDA), food security is defined as access by all people at all times to enough food for an active, healthy life [6]. Food insecurity means that households were, at times, unable to acquire adequate food for one or more household members because they had insufficient money and other resources for food [6]. This measure was established by the USDA using responses from 18 food security questions about food hardships due to financial constraints. Respondents are classified as food insecure if they report three or more food-insecure conditions [6]. Low-income, working families with children have suffered the most during the pandemic, experiencing sharp increases in food insecurity [7]. From March to April 2020, food insecurity doubled in childless households and tripled in households with children, with Hispanic families experiencing the greatest increase in food insecurity [7]. 

In the United States, the three largest food assistance programs include the Supplemental Nutrition Assistance Program (SNAP), the National School Lunch Program, and Women, Infants, and Children (WIC). In 2019, SNAP provided benefits to 35.7 million people in the United States (about 11 percent of individuals), with an average benefit of USD 130 per person per month [6]. The National School Lunch program provided lunches to an average of 29.6 million children each school day in 2019, with 68 percent of the lunches served free, and an additional 6 percent provided at reduced prices [6]. WIC served 6.4 million participants per month at an average monthly cost for food (after rebates to WIC from manufacturers) of about USD 41 per person [6]. 

During the COVID-19 pandemic, relief packages included a 15 percent increase in SNAP’s maximum benefit, amounting to about USD 28 more in SNAP benefits per person per month, or just over USD 100 per month in food assistance for a family of four, with participation increasing between March and April of 2020 by 10 percent (3.6 million) [8,9]. Additionally, lost school meals were replaced through Pandemic-Electronic Benefits Transfer (P-EBT), which provided USD 10.7 billion in benefits from March through September 2020, in which eligible school children receive temporary emergency nutrition benefits loaded on EBT cards that are used to purchase food [9]. The USDA Food and Nutrition Service (FNS) also approved requests from all WIC state agencies to waive select statutory and regulatory requirements, allowing WIC agencies to issue benefits remotely. As a result, participants were not required to collect their WIC benefits in person, were able to enroll or re-enroll in WIC without visiting a clinic in person, and could postpone certain medical tests [9]. 

Although food assistance programs are useful for addressing food insecurity, in 2019 only 43.2 percent of food insecure households received SNAP benefits, only 27.6 percent received free or reduced-price school lunches, and 8.4 percent received WIC food vouchers [6]. Subsequently, as mentioned above, the pandemic increased the number of households experiencing food insecurity. Although SNAP and food assistance programs have been shown to reduce food insecurity, they are not sufficient for many recipients. In addition, price variability during the pandemic impacted their effectiveness [10]. Other strategies, including local farming collectives, neighborhood gardens, increasing access through free delivery and drop-off points, and healthy incentive programs, are necessary to provide food to households at the greatest risk [10]. Healthy food incentive programs, in particular, have been shown to improve food security, increase fruit and vegetable expenditures and intake, and improve healthy eating behaviors [11,12,13,14,15,16,17]. Partnerships between non-profits and grocery retail stores have been shown to be effective at creating incentive programs [16,17,18,19]. Examples of such partnerships include the Utah Double Up Food Bucks program [20], the Market Match Incentive Program in Los Angeles [19], the Target-Wholesome Wave produce prescription partnership [21], and the DC Greens grocery retail produce prescription program [22].

Brighter Bites is a non-profit organization that implements an evidence-based school-based health promotion program in six cities (Houston, Dallas, Austin, New York City, Washington, D.C., and Southwest Florida areas) in the U.S. [23]. During the 2019–2020 school year, 24,263 families were enrolled in Brighter Bites programming. The program operates as a school-based food co-op, distributing fresh produce and nutrition education to low-income children and families in underserved communities to mitigate food insecurity and improve dietary habits [23]. Parent self-report surveys demonstrate that, on average, 70% of families participating in Brighter Bites are reportedly food insecure. During the COVID-19 pandemic, Brighter Bites programming ceased due to statewide school closures and stay-at-home orders [2,24]. The pandemic also resulted in a 22% increase in food insecurity among Brighter Bites families, to 93% reportedly food insecure, and greater than 65% reporting financial hardship and employment uncertainties [25]. In response to this time of crisis, Brighter Bites pivoted rapidly, undertaking a partnership with for-profit grocery retail store companies to provide produce vouchers to Brighter Bites families. The COVID-19 pandemic and related financial crisis was unprecedented and, to our knowledge, there are no published examples in the literature describing the implementation of such novel non-profit–grocery retail partnerships to address food insecurity and provide communities with produce incentives during the initial phase of the pandemic. The purpose of this paper is to describe the produce voucher program, and its implementation, usage, and participant satisfaction among low-income households with children participating in Brighter Bites in four cities in the U.S. 

## 2. Materials and Methods

**Figure 1:**
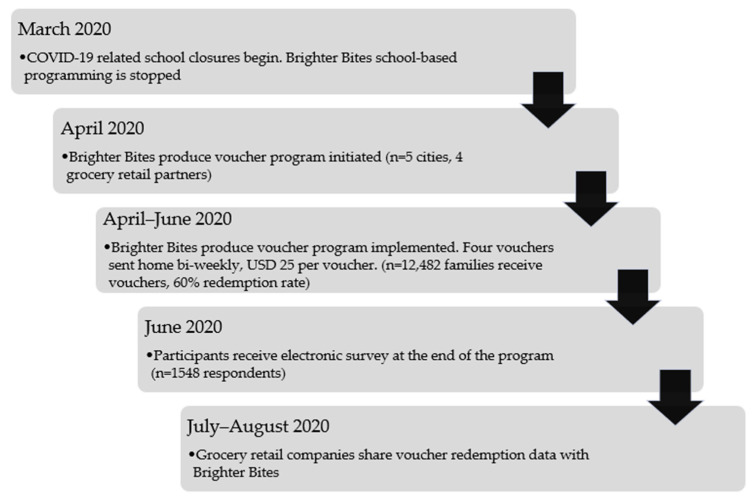
Methodological flowchart of the Brighter Bites produce voucher program implementation.

The Brighter Bites produce voucher program (Figure 1): Subsequent to COVID-19-related school closures in March 2020 in all programming cities, and the resulting increased food insecurity among Brighter Bites families [25], Brighter Bites developed a produce voucher initiative in April 2020 in which each enrolled family received one USD 25 produce voucher bi-weekly for up to four redemptions (USD 100 per family). This new emergency initiative aimed to ensure that all eligible Brighter Bites families had the opportunity to access fresh produce despite school closures as a result of the pandemic. 

Brighter Bites initially rolled out the produce voucher initiative in April 2020 with the Texas grocery retailer H-E-B, in Houston and Austin, Texas, and soon followed in Southwest Florida with Southeastern Grocers, the parent company of Winn-Dixie Stores. Brighter Bites also secured partnerships with a large national chain grocery store in Washington D.C. and 99 Cents Only Stores in Dallas, Texas. 

Implementation: The Brighter Bites voucher program was implemented over nine weeks starting April 2020 with up to four coupons of USD 25 sent to the homes of each participating Brighter Bites family bi-weekly over a nine week period. The vouchers were sent to families by mail. The H-E-B, Winn-Dixie, and 99 Cents Only Stores required the vouchers to be redeemed with a printed coupon due to their technology capabilities. Therefore, Brighter Bites teams reached out to all enrolled families in the participating locations to obtain accurate home addresses to mail the printed coupons. In Houston, Austin, and Southwest Florida (SWFL), vouchers were printed and each of the four coupons were mailed individually. The large national chain grocery store sent vouchers, such that they could be shared electronically via email. 

To redeem the vouchers, families presented them at the participating grocery store at checkout. Vouchers expired 6 weeks after the final voucher was distributed. The voucher program was established such that Brighter Bites paid the grocery retail partner for the cost of the produce subsequent to the voucher redemption. Additionally, each of the grocery retail partners provided a discount on the voucher. The discounts varied by each retail partner. Discount data from H-E-B was unavailable. H-E-B paid for the printing of each voucher and Brighter Bites paid for the postage. Brighter Bites received a 12% discount per voucher at the Winn-Dixie stores in Florida. Winn-Dixie paid for the printing and postage of each voucher. At the 99 Cents Only Stores in Dallas, Brighter Bites received a 20% discount per voucher. The 99 Cents Only Stores paid for printing and postage of each voucher. At the large national chain grocery store in D.C., families received a 25% discount per voucher. There were no printing or postage costs associated with these vouchers because they were sent electronically. However, Brighter Bites paid the large national chain grocery store an initial setup fee of USD 1000 plus USD 0.30 per card. 

Evaluation: Voucher and produce redemption data, including type of produce purchased and amount of money spent on produce, were collected on an ongoing basis by the grocery retail partners and de-identified data was shared with Brighter Bites, and subsequently with UTHealth School of Public Health, for analysis. Brighter Bites has a data use agreement with UTHealth School of Public Health to support program evaluation efforts. The budget impact of the program to Brighter Bites was computed. Participant surveys were conducted by Brighter Bites at the end of the nine week voucher administration period to assess participant utilization and satisfaction of the Brighter Bites voucher program. 

Voucher redemption: At the end of the four redemption periods, the overall number of vouchers redeemed at the city level was provided by the grocery retail partners to Brighter Bites for analysis. 

Produce purchased: Grocery retail partners provided city level data on the type and total amount of produce redeemed at their grocery stores at the end of the voucher redemption period.

Participant Surveys: Survey questions were added to the existing end-of-year Brighter Bites participant process survey to assess voucher access, utilization, ease of use, and usefulness in improving family fruit and vegetable consumption; one open-ended question gauged overall impressions of the voucher program. The self-report process survey was administered electronically in both English and Spanish using Formsite (Vroman Systems Inc., Downers Grove, IL, USA) or paper format, depending on participant preference, to Brighter Bites households who were enrolled by week two of programming in the 2019–2020 school year. Survey completion was voluntary and informed consent was obtained from all participants completing the surveys. One parent/adult family member per household was provided with the survey for evaluation. All data were collected in June–July 2020 by the Brighter Bites non-profit organization as part of their yearly program evaluation efforts. The UTHealth Committee for Protection of Human Subjects Institutional Review Board approved the study protocol and all participants provided written informed consent.

All data were analyzed using Microsoft Excel and STATA 15.0 (STATA Corp, College Station, city, TX, USA). Descriptive statistics, including means, standard deviation, range, frequency, and percent, were computed to determine type of produce purchased, amount of money spent on produce, budget impact of the program, and number of vouchers distributed and redeemed by families, overall and stratified by city.

## 3. Results

Over the course of the nine week program, Brighter Bites distributed a total of over 43,000 vouchers to 12,482 families (52% of Brighter Bites families that responded and provided their addresses). The redemption rate was 60% (at least one voucher redeemed) across all cities. 

Table 1 presents the number of vouchers sent, redemption rate, cost of the program to Brighter Bites and the retail partner for implementation, and total amount of money spent on implementation. Overall, participants collectively made 26,272 visits to the partner retailers, and the overall cost for implementation for Brighter Bites and retail partners was USD 652,295. In Houston, Texas, a total of 29,100 vouchers were sent to families with 17,893 (61%) of those being redeemed. The Brighter Bites cost to implement the program was USD 365,746, and the cost to the retail partner was USD 64,543, resulting in a total program cost of USD 430,290. In Austin, Texas, 8300 coupons were sent to families with 5544 (67%) being redeemed. The Brighter Bites cost in Austin totaled USD 112,998, and the retailer cost totaled USD 19,940, equaling a total program cost of USD 132,938. In Dallas, Texas, 1568 coupons of a total of 2870 (45%) were redeemed, resulting in a total cost of USD 30,393. The Brighter Bites cost totaled USD 25,327, and the retailer cost totaled USD 5066. The Southwest Florida region had the lowest percentage of coupons redeemed, with 967 out of 2332 (41%) being redeemed. The Brighter Bites cost totaled USD 21,274, and the retailer cost equaled USD 2901. The total program cost in Southwest Florida was USD 24,175. Finally, in Washington D.C., 300 of 1380 (22%) coupons were redeemed, totaling USD 34,500 in program costs, with the Brighter Bites cost totaling USD 27,933 and the retailer cost totaling USD 6567. The most selected produce items included peppers, citrus, bananas, tomatoes, berries, avocados, mangoes, onions, and corn.

A descriptive analysis of the usage of the voucher program is presented in Table 2. The survey was completed by 12.2% of families that received the vouchers (n = 1518). Brighter Bites families who responded to the survey comprised 30% English speakers and 70% Spanish speakers, of which 82.9% participated in the produce voucher program. Two-thirds (66.2%; n = 1003) of the respondents who participated in the produce voucher program used 3+ of the produce vouchers provided. Most survey respondents found the vouchers “very easy” (94%) to use and the voucher program “very helpful” (97%) in improving their family’s consumption of produce.

## 4. Discussion

The purpose of the current paper is to describe the implementation and utilization of a novel produce voucher program in which Brighter Bites, a non-profit organization, partnered with multiple large grocery retail partners across five US cities to provide ongoing access to produce during the period of the COVID-19 pandemic when the residents of all five cities were required to shelter-in-place, i.e., April–June 2020. The COVID-19 pandemic has increased the number of families experiencing food insecurity and has worsened conditions for families that had already been food insecure [3,7,26]. Recent studies have reported that in early April 2020, 28% of respondents and 42% of those with children worried about not having enough food [7]. In addition, among Brighter Bites families, studies have demonstrated a 22% increase in food insecurity during the initial shelter-in-place phase of the pandemic (April–June 2020), with fear of COVID-19, unemployment, financial hardship, and food insecurity being the primary concerns among their participating families [25]. In response to this need, Brighter Bites pivoted rapidly to adopting a multi-pronged strategy of (a) increasing their health literacy portfolio [27]; (b) partnering with local food banks and other social service agencies to distribute produce at community sites, and also providing immediate response to families with urgent social needs [28]; (c) partnering with food growers to distribute produce boxes to families as part of the USDA farmers-to-families programing [29]; and (d) forming for-profit–retail partnerships as described in this paper. All strategies were deployed simultaneously in April 2020.

During times of crisis, non-profit–for-profit partnerships, such as the one between Brighter Bites and the grocery retail industry, are feasible and helpful in meeting the needs of the most vulnerable populations, who may not be able to purchase healthy food during these times due to their financial hardship. Providing monetary incentives, such as produce vouchers, is potentially an effective means to improve produce consumption and reduce food insecurity [14,20]. These partnerships can also be mutually beneficial by potentially creating new customers for the retail industry and improving their community relations [30], while promoting healthy eating habits, which is the mission of Brighter Bites. Our study describes the design, implementation, and preliminary findings of the voucher program implementation metrics. Results demonstrated high feasibility of implementation, moderately high redemption rates of the vouchers, and high satisfaction with the voucher program and the produce obtained among those who redeemed them. 

Healthy food incentive programs, such as the USDA Healthy Incentives Program and the Gus Schumacher Nutrition Incentive Program (GusNIP), have been shown to increase fruit and vegetable expenditure and intake [11,12,13], reduce food insecurity [14], and improve healthy eating behaviors [15]. Although effective, these programs are underutilized, with only a small fraction of SNAP participants having access to them [15]. Additionally, consumer demand often outpaces program budgets, and nonfederal financial matching requirements are a burden for state institutions, limiting the scope of incentives [15]. Programs such as the Brighter Bites produce voucher program create innovative partnerships between a non-profit and large grocery retail stores, using both electronic and physical vouchers, administered to low-income families, and allow for the purchase of a variety of produce of their choice. 

Similar to that seen in our study, there are other models of such partnerships in the literature. For example, the Utah Double Up Food Bucks program was a partnership between farmer’s markets, the Utah Department of Public Health, and the non-profits Utahns Against Hunger and Urban Food Connections of Utah. They provided families with USD 10 in the form of tokens as an incentive for purchasing fruits and vegetables at farmers’ markets [20]. Results demonstrated an increased fruit and vegetable consumption and food security among participating families [20]. In Los Angeles, Market Match was a partnership between the non-profit Hunger Action Los Angeles (HALA) and the farmers’ markets, which matched customers’ federal nutrition assistance benefits [19]. Market Match improved food insecurity scores and increased participants’ consumption of produce [19]. Other examples of successful non-profit–company partnerships include the Target-Wholesome Wave partnership, which implemented a produce prescription program that gives families in need “prescriptions” (vouchers) to purchase fruits and vegetables at local Target stores in Los Angeles [21]. Similarly, in Washington D.C., the non-profit DC Greens partnered with Giant Food grocery stores and AmeriHealth Caritas District of Columbia, implementing their own produce prescription program in which food insecure patients screened at the healthcare institution are provided a prescription to redeem produce at the grocery store [22]. In addition to the current study, these studies demonstrate the potential impact of innovative cross-sector partnerships on improving access to, and availability of, social needs among vulnerable populations. Given that food insecurity is likely to be at high levels in the foreseeable future, these partnerships are important to scale and evaluate. 

Strengths of the study include the partnerships across multiple large local and national retail chains, which can potentially allow for rapid scalability of such programs. Furthermore, the data sharing between the retail partners and Brighter Bites allowed for assessment of program feasibility and success of implementation. Limitations include the lack of a stringent study design, including the lack of a comparison group or individual-level dietary intake data, which does not allow for assessment of the impact of the program on child or family produce consumption outcomes. Rather, the current study was an assessment of a natural experiment conducted during the acute phase of the pandemic. Furthermore, analytic approaches, such as social network analysis, may be considered to understand factors that inform the adoption and implementation success of produce voucher programs such as the one implemented here. Other limitations include the availability of data, which varied across the retail partners. In addition, the data was de-identified, which allowed only aggregated findings.

Another limitation is that only about 52% of those enrolled in Brighter Bites received the produce vouchers. This was because only 52% of the Brighter Bites families provided addresses to mail the coupon, despite multiple outreach strategies to families from Brighter Bites staff. Finally, produce vouchers were provided only for four redemptions, totaling USD 100, which may not be sufficient to sustainably address food insecurity. However, Brighter Bites was able to resume programming, including produce distributions, in the summer, using produce boxes distributed to the families.

## 5. Conclusions

The Brighter Bites produce voucher program was widely supported by participating families, easy to use, and valuable in addressing the needs of vulnerable populations who may have been unable to purchase healthy foods during the pandemic due to their financial hardship. This type of partnership has the potential to address critical needs of low-income families by leveraging existing infrastructure across non-profit grassroots efforts such as Brighter Bites, and for-profit grocery retail companies. As a result, these families can be provided with healthy food, particularly during times of crisis, such as the COVID-19 pandemic, when nutrition security is important to maintain health. The produce voucher program had significant social implications for low-income families, including incentivizing the purchase of produce and providing targeted assistance in the form of vouchers to alleviate the food insecurity and financial distress imposed by the pandemic.

Although our study shows promise in the feasibility and acceptability of a produce voucher program, future research is needed to understand the effectiveness of such programs on improving parent and child dietary outcomes, and food insecurity over a longer time period, using a more stringent, experimental study design with a comparison group. 

## Figures and Tables

**Table 1:** Brighter Bites produce voucher program costs and voucher redemption data.

City	Number of Vouchers Sent	Total Number of Vouchers Redeemed N (%)	Brighter Bites Voucher Program Cost (USD) ^§^ (a)	Cost to Retailer (USD) (b) *	Total Program Cost (USD) (a + b)	Most Purchased Produce Items
Houston	29,100	17,893 (61%)	365,746	64,543	430,290	Peppers, Citrus, Bananas, Tomatoes, Berries, Avocados, Mangoes, Onions
Austin	8300	5544 (67%)	112,998	19,940	132,938	Peppers, Citrus, Bananas, Tomatoes, Berries, Avocados, Mangoes, Onions
SWFL	2332	967 (41%)	21,274	2901	24,175	Citrus, Avocados, Berries, Corn, Bananas
Dallas	2870	1568 (45%)	25,327	5066	30,393	N/A
DC	1380	300 (22%)	27,933	6567	34,500	Bananas, Grapes, Mangoes, Tomatoes, Limes, Avocados, Corn, Berries
Totals	43,982	26,272 (60%)	553,278	99,017	652,295	

N/A—produce purchase data not available. ^§^ Cost to Brighter Bites to implement the program; * cost to the retailer to implement the program.

**Table 2:** Voucher satisfaction and utilization from Brighter Bites families participating in the produce voucher program, Brighter Bites process evaluation survey July 2020 (n = 1831).

		Total	
		N	%
Language of survey completion:	English	448	29.5%
	Spanish	1070	70.5%
Did you participate in the BB vouchers/coupons for the purchase of fresh fruits and vegetables? (Choose one)	Yes	1518	82.91%
	No	209	11.41%
	I shared my info, but did not receive vouchers/coupons	104	5.68%
	Total	1831	100.00%
If yes, how many vouchers/coupons have you redeemed at the store? (0,1,2,3,4)	0	13	0.86%
	1	152	10.02%
	2	349	23.01%
	3	550	36.26%
	4	453	29.86%
	Total	1517	100.00%
Did you find the vouchers/coupons easy to use? (Select only one)	Very easy	1425	93.94%
	Somewhat easy	81	5.34%
	Not easy at all	11	0.73%
	Total	1517	100.00%
Did you find the vouchers/coupons program helpful in improving your family’s consumption of produce? (Select only one)	Very helpful	1470	96.84%
	Somewhat helpful	40	2.64%
	Little helpful	7	0.46%
	Not helpful at all	1	0.07%
	Total	1518	100.00%

## Data Availability

The data presented in this study are available on request from the corresponding author.
